# Sickled Erythrocytes Reversal and Membrane Stabilizing Compounds in* Telfairia occidentalis*

**DOI:** 10.1155/2016/1568061

**Published:** 2016-06-28

**Authors:** Samuel Atabo, Ismaila Alhaji Umar, Dorcas Bolanle James, Aisha Indo Mamman

**Affiliations:** ^1^Department of Biochemistry, Ahmadu Bello University, Zaria, Nigeria; ^2^Department of Hematology, Ahmadu Bello University Teaching Hospital, Shika, Zaria, Nigeria

## Abstract

*Background and Purpose*. Traditional management of sickle cell disease (SCD) is ubiquitous in Africa. In south-eastern Nigeria,* Telfairia occidentalis (T. occidentalis)* is strongly recommended for consumption by SCD patients, owing to its presumed therapeutic effect. This study investigates the antisickling and membrane regenerative potentials of* T. occidentalis* in sickled erythrocytes.* Experimental Approach*. Sickled erythrocytes obtained from SCD patients were treated with sodium metabisulphite (2%) to induce further sickling. Heat and hypotonic-induced lyses of red blood cells' membranes were also carried out. The RBCs were treated with varying concentration (10.0, 1.0, and 0.1 mg mL^−1^ and 0.5, 1.0, 1.5, 2.0, and 2.5 mg mL^−1^, resp.) of* T. occidentalis* extracts as treatment regimen for* in vitro* antisickling and membrane stabilizing assays. Extract with peak activity was purified and reused in antisickling assay.* Key Results*. The antisickling activity of aqueous and methanolic extracts of leaves, seeds, and stem of* Telfairia occidentalis* at 10.0, 1.0, and 0.1 mg mL^−1^ revealed that the aqueous leaves extract (10 mg mL^−1^) exhibited the highest antisickling activity (64.03%) which was significantly (*p* < 0.05) higher than that of the stem (47.30%) and seeds (37.50%). Partially purified fractions recorded improved antisickling effect (peak activity of 70%). Characterization (using GC-MS) of the most active fraction revealed some bioactive compounds. In the membrane stabilizing assay, methanolic and aqueous stem extracts of* T. occidentalis* showed the highest effect of 71.85% and 61.29%, respectively.* Conclusions and Implications*. The results provide scientific evidence for ethnopharmacological use of* T. occidentalis* in the management of SCD.

## 1. Introduction

Sickle cell disease (SCD) is a neglected tropical disease [1], and its global burden has continued to increase in low- and middle-income countries [2], especially in sub-Saharan Africa. It is a devastating genetic disorder affecting 2.3% of the world population, mainly in Africa [3], and responsible for about 2% death in children below the age of 5 in sub-Sahara Africa [3]. In Nigeria, prevalence of SCA is about 20–30 per 1000 births [3], and in June 19, 2012, the World Health Organization (WHO) reported that Nigeria has the highest number of sickle cell anaemia sufferers worldwide [4].

Various methods have been developed in an effort to find principles that prevent or reduce crises in SCD. Conventional medicines have achieved a great level of success in SCD management. In recent years, bone marrow transplantation has been found to be efficient in the treatment of SCD. However, the cost implications, unavailability of necessary expertise, and the problems of finding suitable donor constitute a major setback to this approach in developing countries [5]. In clinical practice, blood transfusion, Hydroxyurea, Hydroxycarbamide, Clotrimazole, and erythropoietin are used in SCD management, but the side effects of these drugs such as iron overload and mutagenic and teratogenic assertions limit their clinical use [6, 7].

On the understanding that herbal remedies and medicinal plant products from indigenous flora have long been used in traditional medicine for SCD management, it appears that proper and intense scientific investigations on these medicinal plants could be of remarkable help in developing effective and safer drugs for SCD treatment. Research on phytomedicine has led to the development of Nicosan (herbal based drug used to treat SCD). Other plants that have gained scientific backing as antisickling agents include* Cajanus cajan* [8, 9],* Carica papaya* [10–12], and* Fagara zanthoxyloides* [13, 14].

*Telfairia occidentalis* which has been linked to free radical scavenging, boosting of blood levels, and antiplasmodial and antimicrobial effects [15–17], is now being speculated in this study as a possible therapeutic agent for SCD management, owing to its consumption by SCD patients in south-eastern Nigeria.

## 2. Materials and Methods

### 2.1. Equipment, Chemicals, and Reagents

Some equipment used included Shimadzu GC-17A system, light microscope (Olympus CX 21), spectrophotometer (Jenway 6400), incubator (grant JB series), oven (Gallenkamp), and centrifuge (Labofuge 300). Chemicals such as Para-hydroxybenzoic acid (PABA) and sodium metabisulphite were procured from Sigma Chemical Company, Paderborn, Germany.

### 2.2. Collection and Extraction of Plant Materials

Fresh samples of* Telfairia occidentalis* were purchased from a farm in Kagarko local government area of Kaduna state. The plant was identified and authenticated at the Herbarium Unit, Biological Science Department, Ahmadu Bello University, Zaria, with a voucher specimen number 3252. Leaves, seeds, and stem of the plant were collected, shade-dried, and powdered using mortar and pestle. Each of the dried powdered material (500 g) was extracted with 2 L of methanol by cold maceration for 7 days in large amber bottles with intermittent shaking. At the end of the extraction, the crude methanolic extract was filtered using Whatman filter paper number 42. The filtrate was concentrated by evaporation (using water bath at 45°C). In another setup, dried powdered materials were soaked in distilled water for three days using the same proportion used for methanol extraction. It was overlaid with chloroform to prevent fermentation and bacterial degradation by inhibiting possible oxygen infiltration (i.e., H_2_O : CHCl_3_, 70 : 30). This was followed by evaporation to dryness to obtain aqueous extract. Filtrates obtained in both cases were reconstituted in normal saline.

### 2.3. Blood Collection, Ethical Approval, and Informed Consent

Blood (2 mL) was obtained by venipuncture from homozygous sickle cell disease (HbSS) patients in stable state at Ahmadu Bello University Teaching Hospital (ABUTH), Zaria. Approval by the Health Research Ethical Committee (HREC) and consent of participants were obtained in accordance with the ethical standards laid down in the 1964 Declaration of Helsinki.

### 2.4. Identification of Homozygous Sickle Cell Disease Patients

Differentiation of major genotypes method by hemoglobin electrophoresis technique was used.

The principle of hemoglobin electrophoresis (at alkaline pH 8.4–8.9) was based on charge change in hemoglobin molecules, which correlates with the rate of hemoglobin movement towards the anode.

Blood samples of different, but known, genotypes were spotted at the point of origin on the paper. It was then placed in the electrophoretic component containing a buffer system and connected to a source of current. Hemoglobin movement towards the anode (represented by hemoglobin bands) was peculiar to each genotype (HbAA, HbAS, HbSS, and HbSC). This was noted and was used as standard. Subsequent test samples were treated as mentioned above. Those that showed similar pattern of movement as HbSS (when compared with the standard) were obtained and used in this research.

### 2.5. Blood Preparation

The blood was placed in sodium ethylene diamine tetraacetic acid (EDTA) bottle and thoroughly mixed by gently rolling the bottle. Blood samples were used for the studies within 12 hrs of collection.

### 2.6. Sample Size Determination

The minimum sample size in this study is determined by the formula of [18]:

(1) $$\begin{array}{c} n=\frac{Z^{2}pq}{d^{2}}, \end{array}$$

where *n* is the minimum sample size, *Z* is the value of the normal curve corresponding to 95% confidence interval, 1.96, *p* is the prevalence of sickle cell disease, 2.3%, *q* = 1 − *p*, that is, 1 − 0.023 = 0.977, *d* is the level of significance or error margin, 5%, and *n* = 1.96^2^ × 0.023 × 0.977/0.05^2^ = 0.088/0.0025 = 35.2; therefore, the minimum sample size was approximately 35.

### 2.7. Bioassay of Plant Extracts for Antisickling Activity

The bioassay of both methanolic and aqueous extracts of the plant materials for antisickling activity was carried out by measuring the level of reversal of sickled red blood cells (RBCs).

### 2.8. Evaluation of Antisickling Activity

Evaluation of antisickling activity was carried out according to the procedure of [19].

#### 2.8.1. Principle

Washed erythrocytes were mixed with 2% sodium metabisulphite. Sodium metabisulphite initiates a decrease in oxygen tension thereby inducing the typical sickle shape of red blood cells. This is then followed by introduction of test extract in the test tube. The degree of reversal of sickling was measured by counting the number of cells unsickled under the light microscope.

#### 2.8.2. Procedure

Half a milliliter (0.5 mL) of the washed erythrocytes was mixed with 0.5 mL of freshly prepared 2% sodium metabisulphite in a clean test tube. It was incubated in water bath at 37°C for 30 minutes. A drop of the mixture was then viewed under the microscope. Equal volumes (0.5 mL) each of normal saline and the extracts were added to the blood-metabisulphite mixtures in different test tube and incubated at 37°C for another 30 minutes. Aliquots were taken at 30 minutes intervals, for up to 2 hours.

**Figure 1:**
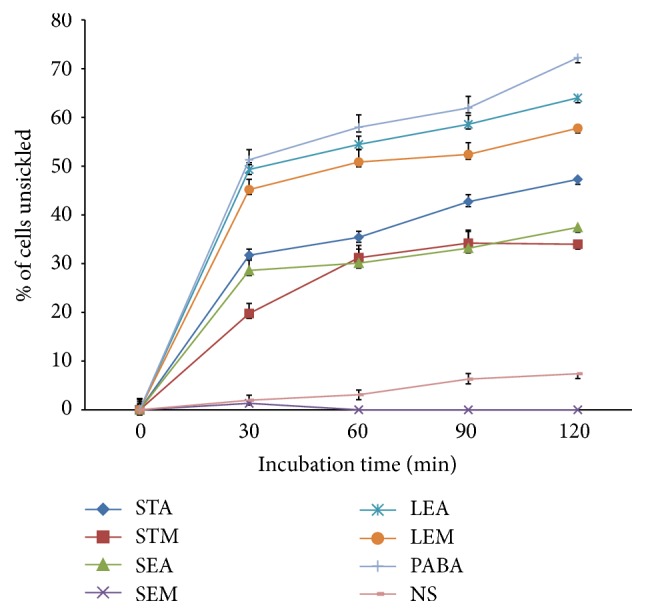
Percentage cells unsickled with time by 10 mg mL^−1^ extracts of leaves, seeds, and stem. LEA: aqueous leaves extract, LEM: methanolic leaves extract, SEA: aqueous seed extract, SEM: methanolic seeds extract, STA: aqueous stem extract, STM: methanolic stem extract, NS: normal saline, and PABA: Para-hydroxybenzoic acid.

The procedure described by [20] was used for smear preparation and counting of sickled and unsickled cells. Briefly, each sample was smeared on microscope slide, fixed with 95% methanol, dried, and stained with Giemsa stain. It was then examined under an oil immersion microscope and counting red blood cells was carried out by viewing from different fields (4 fields) across the slide. The numbers of both sickled and unsickled red blood cells were counted and the percentage of cells unsickled was determined (Figure 1).

It was calculated by the following formula:

(2) Percentage  of  cells  unsickled=Number  of  cells  unsickledTotal  number  of  sickled  cells×100.

### 2.9. Antisickling Experimental Control

P-Hydroxybenzoic acid (5 mg mL^−1^) and normal saline were, respectively, employed as positive and negative controls.

### 2.10. Membrane Stabilizing Activity

The membrane stabilizing assay method was based on the procedure described by [21].

The assay mixture consisted of 2 mL of 0.25% (w/v) NaCl, 1.0 mL of 0.15 M sodium phosphate buffer (pH 7.4), 0.75 mL of various concentrations (0.5, 1.0, 1.5, 2.0, and 2.5 mg mL^−1^) of plant extracts, and 0.5 mL of (2% v/v) erythrocyte suspension. The control was prepared as mentioned above, but without drug or extract (blood control), while the drug control was without extract. The standard drugs used were Indomethacin and Ibuprofen. The reaction mixtures were incubated at 56°C for 30 minutes, cooled under running water, and then centrifuged at 3913 ×g.

The principle behind this assay is the spectrophotometric measurement of the amount of hemoglobin released (read at 560 nm) by sickled erythrocytes, which is dependent on the extent of stabilization of sickled red blood cells' membrane exerted by the test extract.

The percentage membrane stability was estimated thus:

(3) %  Membrane  stability=100−absorbance  drugtest−absorbance  drugcontrol×100Absorbance  bloodcontrol.

### 2.11. Thin Layer Chromatography (TLC)

Commercially prepared TLC aluminum sheets (20 cm by 20 cm) lined with silica gel were used. The plate was cut to fitted size of 5 × 5 cm. The extract was dissolved in 95% methanol and spotted at the bottom of the TLC plate (about 0.5 cm from the base) using a micro haematocrit capillary tube. The plate was placed in a chromatographic tank and eluted with a mixture of different solvents. The gradient mixture of hexane and ethyl acetate mixture gave the best resolution. Hexane/ethyl acetate at different ratios (100% hexane 9 : 1 v/v, 8 : 2 v/v, 7 : 3 v/v, 6 : 4 v/v, 5 : 5 v/v, 4 : 6 v/v, 3 : 7 v/v, 2 : 8 v/v, 1 : 9 v/v, and 100% ethyl acetate) was used. The plate was then removed, air-dried, and developed by spraying with 10% sulphuric acid in methanol. It was viewed under UV light. Spots, where seen, hence adjudged the solvent system of choice for use in column chromatography.

### 2.12. Partial Purification of Crude Extract (Column Chromatography)

Partial purification of active crude extracts using a modified method of [22] was employed. In this method, the crude extract of* T. occidentalis* was separated using silica gel (as adsorbent, 70–230 mesh) packed in a chromatographic column. The adsorbent was overlaid with cotton wool before and after the application of plant extract. The cotton wool is to prevent direct contact or mixing of extract with the silica gel, to absorb shock (that may cause crack in the loaded adsorbent) when solvent is being poured into the chromatographic column, and to serve as filter for particles that may lurk within the solvent. Slurry of powdered silica gel in hexane was packed in glass column (50 cm) to a height of about 12 cm (and diameter of 3 cm). It was then loaded with 10 mL of extracts dissolved in methanol and separated by gradient elution with solvent system of choice (100% hexane 9 : 1 v/v, 8 : 2 v/v, 7 : 3 v/v, 6 : 4 v/v, 5 : 5 v/v, 4 : 6 v/v, 3 : 7 v/v, 2 : 8 v/v, 1 : 9 v/v, and 100% ethyl acetate) at different proportion and in order of their increasing polarity. Fractions (10 mL) were collected into different labeled beakers. The solvent was allowed to evaporate and the content spotted on TLC chromatographic sheet to view the level of resolution on the basis of spots. Fractions that showed the same number of spots and the same colour and retention factor (*R*_*f*_) values were pulled together and used for antisickling assay.

### 2.13. Characterization of Purified Fraction

The purified fraction that showed peak antisickling activity was characterized using Gas Chromatography linked Mass Spectroscopy (GC-MS). The fraction was dissolved in methanol and applied on a Gas Chromatography (GC). It was separated at 60°C at a flow rate of 1.83 mL/min, 109.6 Kpa pressure for 4.15 min. Eluents were bombarded with a stream of protons from mass spectra (MS) and analyzed.

### 2.14. Statistical Analysis

Results were presented as mean ± standard deviation (SD). Within and between the groups, comparisons were performed by analysis of variance (ANOVA) (using SPSS 17.0 computer software package). Significant differences were compared using Duncan Multiple Range Test (DMRT), and a probability level of less than 5% (*p* < 0.05) was considered significant.

## 3. Results

### 3.1. *In Vitro* Antisickling Effects of Aqueous and Methanolic Extracts of Leaves, Seeds, and Stem of* Telfairia occidentalis*

Data on* in vitro* studies of the antisickling activity of* T. occidentalis* extracts carried out on blood samples collected from noncrisis sickle cell patients showed reversal of sickle cells at different rates and at different extract concentrations (Table 1). The leaves extracts (aqueous and methanolic) at 10 mg mL^−1^ exhibited the highest unsickling activity of 64.03%  ±  1.69 and 57.79%  ±  2.61, respectively, compared to the positive control (p-hydroxybenzoic acid) which showed activity of up to 72.25%  ±  1.85.

Aqueous and methanolic crude extracts of the stem demonstrated a dose- and time-dependent increase in reverting sickled cells back to normal. However, the methanolic extract of seeds triggered partial lysis of erythrocytes at the maximum concentration of 10 mg mL^−1^, after 30 minutes of incubation. Complete lysis was seen between 60 and 120 minutes, by the effect of 10 mg mL^−1^ of methanolic seeds extract. This may imply that, at high concentration and over time, seeds extract of* Telfairia occidentalis* may have a cytotoxic effect (Table 1).

### 3.2. Membrane Stabilizing Effect of Extracts from the Different Plant Parts

Table 2 provides the membrane stabilizing activity of all fractions. Both aqueous and methanolic extracts of leaves and seeds of* T. occidentalis* exhibited a maximum inhibition (to haemolysis) of 27.05% and 32.24%, respectively. The methanolic extract of the stem showed a more promising membrane stabilizing activity of 71.85% when compared with other fractions. The membrane stabilization activities of the standard drugs, Ibuprofen and Indomethacin, were 62.45% and 81.82%, respectively.

### 3.3. Partial Fractionation of Active Extract with Antisickling Activity

#### 3.3.1. Results of Column Chromatography Cum Thin Layer Chromatography

Sixty (60) fractions were obtained from column chromatography of the most active crude extract (aqueous leaves extract). A gradient mixture of hexane and ethyl acetate was used as eluent. Fractions with the same number of spots, *R*_*f*_ values, and the same colour under UV light were pooled together and used to test for antisickling activity.

### 3.4. GC-MS Analysis

The GC-MS analysis of purified fraction that showed peak antisickling activity (Fraction C) revealed some associated bioactive components. Their molecular weight, chemical formula and retention index are shown in Table 4.

## 4. Discussion

Results obtained from this research indicated that* in vitro* antisickling action of extracts (leaves, stem, and seeds), especially the aqueous leaves extract, was rapid. A 64.03% reversal of sickle cells was observed in 120 minutes by the effect of the crude leaves extract of* T. occidentalis*. Partial purification (fractionation) of leaves extract exhibited a distinctive increase in antisickling activity, with fraction C (Table 3) showing the highest level of reversal of sickled erythrocytes (74%). The higher antisickling activity shown by the partially purified extract is attributed to the fact that bulky interfering molecules such as impurities, artifacts, and contaminants have been eliminated by the purification process, thereby leaving behind the pure bioactive components.

Bioactive compounds such as *α*-phellandrene, borneol, 16-octadecenoic acid, 9-octadecadienoic acid (Z)-, 2,3-dihydroxypropyl ester, pentadecanoic acid, 14-methyl ester, and *α*-campholene were elucidated using Gas Chromatography Linked Mass Spectroscopy (GC-MS) (Table 4).

Alpha-Phellandrene (*α*-phellandrene) has been reported to inhibit inflammation [23], promote immune response [24], and fight infections [25]. Also, borneol, pentadecanoic acid, 16-octadecenoic acid, 9-octadecadienoic acid (Z)-, 2,3-dihydroxypropyl ester, and *α*-campholene aldehyde have been linked with analgesic, antibacterial, antioxidant, antipyretic, antispasmodic, and sedative activity [26]. The aforementioned conditions are central to sickle cell disease crisis, thereby suggestive of the fact that* T. occidentalis* may presage a therapeutic potential in SCD. If this action can be reproduced* in vivo*, then the extract may as well hold a lot of promise in the treatment of sickle cell disease.

Depending on its half-life, it would be expected that its periodic administration would reduce both frequency and duration of crises.

Pathophysiology of sickle cell disease has been attributed to both sickle hemoglobin and erythrocyte-membrane behavior [27]. Membrane stabilizing effect of various concentrations of extracts (leaves, stem, and seeds) on sickle red blood cells revealed an overall maximum activity of 71.85 ± 0.001% in the stem methanolic extract and minimum activity of 22.60 ± 0.003% in aqueous leaves extract. The results showed that both extracts contained principles that protected the erythrocyte membrane effectively. Membrane stabilizing activity was observed to be plant part and concentration-dependent. Also, the aqueous leaves extract showed the least protection on erythrocyte membrane.

On the basis of these results, it could be inferred that the aqueous and methanolic extracts of leaves, stem, and seeds of* T. occidentalis* contained principles that were capable of stabilizing sickle erythrocyte membrane against heat and hypotonic-induced lysis.

## 5. Conclusion

The results of this study scientifically validated the* in vitro* potential of* T. occidentalis* in the management of sickle cell disease.

## Figures and Tables

**Table 1:** Peak antisickling effect of crude aqueous and methanolic extracts of *Telferia occidentalis* (fluted pumpkin) leaves, seeds, and stem.

Crude extract	% of cells unsickled at 120 min		
	10 mg	1 mg	0.1 mg
LEA	64.03 ± 1.69^c,5^	60.59 ± 1.60^b,5^	55.45 ± 1.46^a,5^
LEM	57.79 ± 2.61^c4^	50.82 ± 1.18^b,4^	46.11 ± 1.72^a,4^
STA	47.33 ± 2.11^b,3^	43.69 ± 3.20^b,3^	32.79 ± 1.80^a,3^
STM	33.98 ± 1.54^c,1^	26.51 ± 1.52^b,1^	15.36 ± 0.41^a,1^
SEA	37.45 ± 1.16^c,2^	31.35 ± 1.20^b,2^	27.48 ± 1.23^a,2^
SEM	—	—	36.23 ± 3.70

Values in the same row with different superscripts (a–c) are significantly different at *p* < 0.05.

Values in the same column with different superscripts (1–5) are significantly different at *p* < 0.05.

LEA: leaves aqueous extract, LEM: leaves methanolic extract, STA: stem aqueous extract, STM: stem methanolic extract, SEA: seeds aqueous extract, and SEM: seed methanolic extract.

Note: “—” represents complete lysis of hemoglobin.

**Table 2:** Membrane stabilizing activity of *Telferia occidentalis* fractions.

Extracts	Concentration (mg/mL)				
	0.5	1.0	1.5	2.0	2.5
LEA	3.13 ± 1.69	9.41 ± 0.86	15.81 ± 2.81	20.13 ± 0.59	23.49 ± 3.11
LEM	6.29 ± 1.84	14.94 ± 2.56	20.93 ± 1.86	25.42 ± 0.10	26.80 ± 0.25
STA	28.41 ± 4.31	43.67 ± 1.28	50.17 ± 2.16	55.67 ± 3.21	60.14 ± 1.15
STM	44.64 ± 2.19	53.22 ± 4.33	62.12 ± 0.88	66.00 ± 2.11	69.31 ± 2.54
SEA	3.92 ± 1.31	12.16 ± 1.77	15.04 ± 2.26	25.21 ± 1.07	29.02 ± 1.28
SEM	7.87 ± 2.62	19.03 ± 1.89	26.42 ± 1.78	30.17 ± 1.22	31.05 ± 1.19
Indomethacin	30.21 ± 1.07	44.56 ± 0.18	64.13 ± 3.01	70.02 ± 0.92	78.94 ± 2.88
Ibuprofen	26.26 ± 1.99	35.81 ± 3.42	49.38 ± 1.43	58.85 ± 2.32	61.33 ± 1.12

LEA: leaves aqueous extract, LEM: leaves methanolic extract, STA: stem aqueous extract, STM: stem methanolic extract, SEA: seeds aqueous extract, and SEM: seed methanolic extract.

**Table 3:** Peak antisickling effect of partially purified fractions of crude aqueous leaves extracts of *Telfairia occidentalis* (fluted pumpkin).

Partially purified fractions	Percentage (%) of cells unsickled at 120 min		
	10 mg	1 mg	0.1 mg
Fraction B	55 ± 2.92	51 ± 1.61	43 ± 1.75
Fraction C	70 ± 3.39	62 ± 2.84	54 ± 1.56
Fraction D	65 ± 2.66	56 ± 2.53	44 ± 3.44
Fraction E	52 ± 1.46	48 ± 1.18	41 ± 4.89

PABA (5 mg mL^−1^)	74 ± 3.88		
Normal saline	7 ± 0.69		

**Table 4:** GC-MS analysis of fraction C.

S/no	Name of compound	Chemical formula	M. wt.	RI
(1)	Pentadecanoic acid, 14-methyl ester	C_17_H_34_O_2_	270	619
(2)	Octadecanoic acid	C_18_H_36_O_2_	283	749
(3)	Hexadecanoic acid	C_16_H_32_O_2_	256	648
(4)	9-Octadecadienoic acid (Z)-, 2,3-dihydroxypropyl ester	C_21_H_40_O_4_	356	756
(5)	*α*-Campholene aldehyde	C_19_H_16_O	152	1123
(6)	Cyclohexanespiro-5′,4′-methyl-2′-phenyl-2′-oxazoline	C_15_H_19_NO	229	947
(7)	*α*-Phellandrene	C_10_H_16_	136	1243
(8)	16-Octadecenoic acid	C_19_H_36_O_2_	296	718
(9)	Borneol	C_10_H_18_O	154	1164

## Abbreviations

SCD: Sickle cell disease
TLC: Thin Layer Chromatography
*R*_*f*_: Retention factor
ANOVA: Analysis of variance
DMRT: Duncan Multiple Range Test
HbSS: Homozygous sickle cell disease
HREC: Health Research Ethical Committee
RBC's: Red blood cells
LEA: Aqueous leaves extract
LEM: Methanolic leaves extract
SEA: Aqueous seed extract
SEM: Methanolic seed extract
STA: Aqueous stem extract
STM: Methanolic stem extract and Para-hydroxybenzoic acid (PABA).
